# Relating D-Dimer, blood sugars, haemoglobin and liver function among COVID patients with T2DM

**DOI:** 10.6026/973206300191167

**Published:** 2023-12-31

**Authors:** Balaji Viswanatha Setty, Gutlur Nagarajaiah Setty Raghav, Hareesh Rangaswamaiah

**Affiliations:** 1 Department of General Medicine, Rajarajeshwari Medical College and Hospital, Bangalore, Karnataka, India

**Keywords:** COVID 19, D-Dimer, T2DM, cycle threshold

## Abstract

It is of interest to evaluate the correlation of D-Dimer, blood sugars, haemoglobin and liver function tests with novel coronavirus in patients with T2DM with and without symptoms. We recruited 200 patients with T2DM and COVID 19 with and without symptoms admitted in Rajarajeshwari Medical College and Hospital, Karnataka. Blood sugars, HbA1c, D-Dimer and also the incidence of T2DM and COVID 19 with and without symptoms were evaluated in all study subjects. There was a significant increased levels of biochemical parameters in T2DM and COVID 19 with symptoms when compared to T2DM and COVID 19 without symptoms (P<0.05). The D-Dimer levels was positively correlated with CT values, (r=0.518, P<0.05). Based on the study findings, the novel coronavirus enhances the insulin resistance, hyper-glycemia, abnormality in the liver and thrombolysis. Additionally, we also suggest that the subjects with T2DM and COVID 19 with and without symptoms require continuous monitoring of D-DIMER and LFT.

## Background:

The severe acute respiratory syndrome coronavirus 2 is caused by the novel coronavirus 2 in the year 2019. It was first identified in December 2019; Wuhan, China later on it spreads throughout worldwide. According to World Health Organization, 5,488,825 peoples were affected and 349,095 peoples are died due to novel coronavirus 2, till May 2020. The prevalence is continuously increasing to 44,351,506 people were affected and 1, 71, 255 peoples were died with SARS-COVID 2 as reported till October 2020 [1-2]. Both symptomatic and asymptomatic clinical manifestations lead to chronic respiratory problems and it also affect other organs like heart, kidney, liver, stomach, and intestine 9 [3]. The subjects with adults, old age group, males, and whoever having other illness such as obesity, diabetes mellitus, kidney diseases, liver diseases, thyroid disorders, cardio vascular diseases and cerebrovascular diseases were high risk of SARS-COVID 2 [4]. Recent research studies are reported that, the novel SARS-COVID 19 affects the pancreas results insulin resistance leads to diabetes mellitus. The endocrine part of pancreas damaged by novel SARS-COVID 2 results improper secretion and activation of insulin cause insulin resistance [5- 6]. The insulin resistance is major cause to disturb carbohydrate and fat metabolism leads to hyperglycemia and type 2 diabetes mellitus. Some other studies are reported that type 2 diabetes mellitus is also considered one of the major risk factors to get the novel SARS-COVID 19 and progressively death also will occur. Hyper-glycemia stimulates the production of reactive oxygen species, this leads to oxidative stress, endothelial damage, inflammation and thrombolytic effects [7]. The fibrinogen breakdown product is D-dimer is a by-product of fibrin breakdown, contributes to thrombo-inflammation in COVID-19. Numerous studies have linked higher D-dimer levels to worsening COVID-19 symptoms and outcomes. Along with that there is a need to evaluate the role of D-Dimer for progression of novel COVID 19 disease in subjects with type 2 diabetes mellitus [8-9]. Similarly, other recent reports found the subjects with COVID 19 are more prone to get liver functions abnormalities [10- 11]. Therefore, it is of interest to evaluate the correlation of D-Dimer, blood sugars, haemoglobin and liver function tests with novel coronavirus in patients with T2DM with and without symptoms.

## Materials and Methods:

This is an observational study conducted in "Rajarajeshwari Medical College and Hospital" over period of 1 year from November 2021 to December 2022. We included 200 consecutive patients attended to medicine OPD and diagnosed with novel Severe Acute Respiratory Syndrome Coronavirus Disease 19. These cases were sub-classified into 2 groups, i.e., 100 SARS-COVID 19 Patients with Asymptomatic were considered as Group 1 and remaining 100 were SARS-COVID 19 Patients with symptomatic were considered as Group 2. The patients were recruited after obtaining approval from Institutional Ethics Committee (IEC No: AIMSRC/IEC 564/2021-2022) and properly filled consent from the patients.

## Criteria of the Study:

## Inclusion Criteria:

All the subjects' age should be 30 to 70 years and diagnosed with type 2 diabetes mellitus (T2DM). All the study subjects are tested COVID 19 quantitatively and CT values should be less than the 35. Along with that the cases were sub grouped into 2 groups, Group 1: T2DM and SARS-COVID 19 patients without symptoms and Group 2: T2DM and SARS -COVID 19 with classical symptoms like cold, sore throat, cough, body pains and fever.

## Exclusion Criteria:

The subjects with having smoking, alcoholism, non SARS-COVID 19, other types of diabetic subjects, hypertension, acute and chronic infections, deep vein thrombosis, pulmonary embolism, kidney diseases, neoplastic diseases, liver diseases, thyroid diseases, cardiovascular diseases, cerebrovascular diseases, peripheral vascular disorders and those are not interested to participate in this study were excluded.

## Sample Collection:

We collected nasopharyngeal and oro-pharyngeal samples were collected with the individual swabs and put it into the viral transport media (VTMs). All the samples were transferred to molecular laboratory for COVID 19 testing quantitatively. Later on, from selected subjects, we collected 12 to 14 hours overnight 10 ml of fasting blood sample and 2ml transferred into anticoagulant and anti-glycolytic vacutainer (Sodium Fluoride), 2ml of blood transferred into Ethylene Diamine Tetra Acetic acid vacutainer, another 3ml transferred into Sodium Citrate vacutainer and remaining 3 ml transferred into plain tube. Again, 2ml post prandial blood sample collected from all the study subjects after 2 hours of breakfast transferred into anti-coagulant and anti-glycolytic vacutainer (Sodium Fluoride). All the vacutainers separated by the process of centrifugation and the plasma and serum immediately analyzed blood sugars, HbA1c, LFT and D-DIMER.

## Methods:

The Fasting Blood Sugars (FBS), Post Prandial Blood Sugar (PPBS) was analyzed by glucose oxidase and peroxidase method, HbA1c determined by latex immunoassay method, Total Bilirubin and Direct Bilirubin was analyzed by using Diazo Method, AST, ALT, ALP was determined by laboratory standard methods, Total Protein was analyzed by biuret method and albumin was determined by bromocresol green method. D-DIMER was measured by using immunofluorescence method.

## Statistical Analysis:

The continuous data was represented as mean ± standard deviation. The one way analysis of variance (ANOVA) was used to test comparison between the variables and groups. To correlate between the variables by Pearson correlation analysis was done. The Microsoft excel spread sheet and statistical package for the social software's used to do all the statistics. A probability (P) values is less than 0.05 was considered as statistically significant. The mean ± SD of FBS in the T2DM and SARS-COVID 19 without symptoms and T2DM and SARS-COVID 19 with symptoms was found to be significant (P<0.05) and PPBS not shown any significant between the study subjects, respectively P value is 0.486. The mean ± SD of HbA1c, CT, D-Dimer, Total Bilirubin, Direct Bilirubin, AST, ALT, ALP in the T2DM and SARS-COVID 19 without symptoms and T2DM and SARS-COVID 19 with symptoms was found to be increasing the significant values is P<0.05. The mean ± SD of Total Protein and in the T2DM and SARS-COVID 19 without symptoms and T2DM and SARS-COVID 19 with symptoms was found to decreased the significant value is P<0.05 (Table 1). Table 2 illustrates the Pearson correlation between CT and other study parameters of the study. The CT was found to have positive correlation with HbA1c, D-Dimer, Hb, Total Bilirubin, Direct Bilirubin, AST, ALT, and ALP, whereas FBS and Albumin showed a significant negative correlation, respectively P value is less than 0.05. The PPBS and Total Protein does not show any significance with CT the P values are respectively 0.43 and 0.53. Table 3 gives the Pearson correlation between D-Dimer and other study parameters of the study. The D-Dimer was found to have positive correlation with PPBS, HbA1c, CT, Total Bilirubin, Direct Bilirubin, AST, ALT, and ALP, whereas FBS, Hb, Total Protein and Albumin showed a significant negative correlation, respectively P value is less than 0.05. Table 4 illustrates the Pearson correlation between D-Dimer and other study parameters of the study. The D-Dimer was found to have positive correlation with HbA1c, CT, Total Bilirubin, Direct Bilirubin, AST, ALT, and ALP, whereas FBS, D-Dimer, Total Protein and Albumin showed a significant negative correlation, respectively P value is less than 0.05. The PPBS and HbA1c does not show any significance with Hb respectively P values are 0.41 and 0.12.

**Figure 1:**
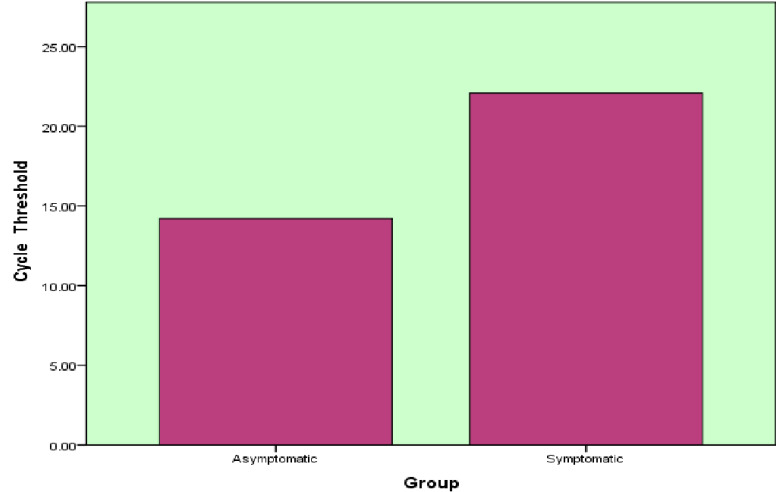
Box plots of CT levels in T2DM and COVID 19 with and without symptoms

Figure 1 shows the Cycle Threshold values of both T2DM AND SARS-COVID 19 with and without symptoms. There was a significantly very low levels of CT values are shown in T2DM and SARS-COVID 19 with symptomatic when compared to T2DM and SARS-COVID 19 without symptomatic patients.

**Figure 2:**
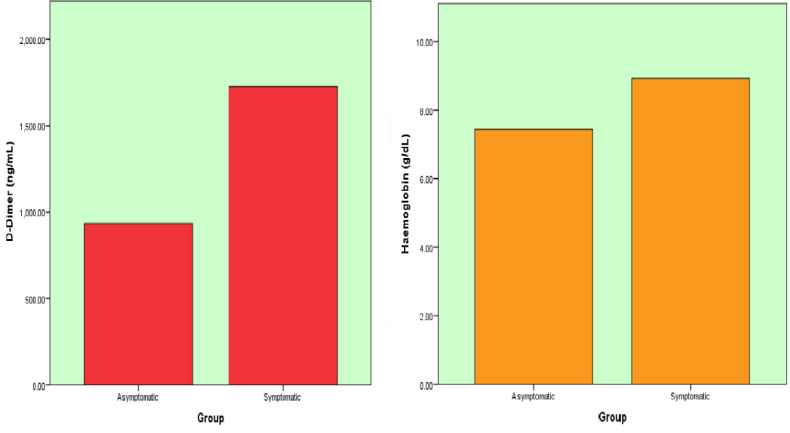
Box plots of D-Dimer, Hb levels in T2DM and COVID 19 with and without symptoms

**Figure 3:**
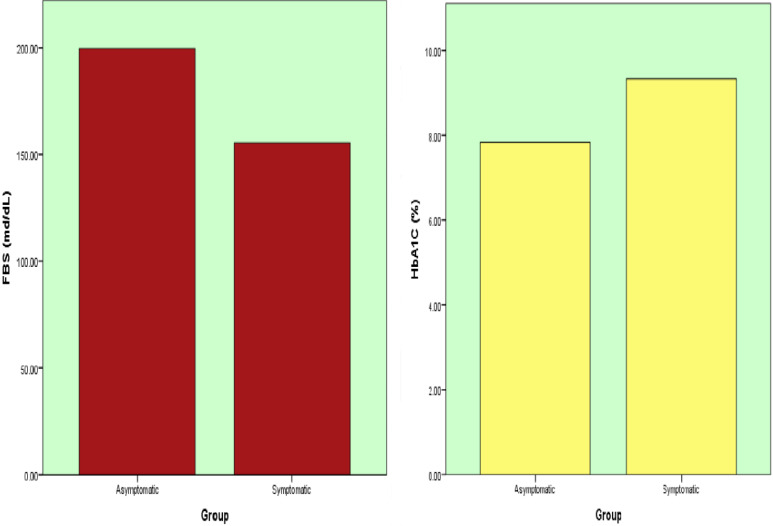
Box plots of FBS, HbA1c in T2DM and COVID 19 with and without symptoms

Figure 2 shows the D-Dimer and Hb values of both T2DM AND SARS-COVID 19 with and without symptoms. The D-Dimer showed significantly very high levels in T2DM and SARS-COVID 19 with symptomatic when compared to T2DM and SARS-COVID 19 without symptomatic patients. Figure 3 shows the fasting blood sugars and HbA1c values of both T2DM AND SARS-COVID 19 with and without symptoms. The fasting blood sugars significantly increased in subjects with T2DM AND SARS-COVID 19 without symptoms than the T2DM AND SARS-COVID 19 with symptoms. The HbA1c significantly increased in subjects with T2DM AND SARS-COVID 19 with symptoms than the T2DM AND SARS-COVID 19 without symptoms.

## Discussion:

The severe acute respiratory syndrome coronavirus 2 is an inflammatory disease in 2019, it become outbreak and global pandemic. Therefore, it is still vital and necessary to identify the independent predictors of COVID-19 mortality in order to decrease the undesirable results. Two intrinsically fibrinogen D domains combined make up the D-dimer, a fibrinogen disintegrate product that reflects strong coagulation and enhanced secondary fibrinolytic activity in vivo [12- 13]. Recent research studies are reported there was a significant association between the D-Dimer and SARS-COVID 19 [14-15]. Endothelial cells may become dysfunctional as a result of hyper inflammation and hypoxia-induced damage brought on by SARS-CoV-2 infection, which may also promote thrombosis and raise D-dimer levels. Deep venous thrombosis, disseminated intravascular coagulopathy, and pulmonary micro-thrombus could all develop as a result of elevated D-dimer, and these conditions were linked to a bad prognosis [16]. Some of the studies are reported there was a strong relation between type 2 diabetes mellitus and SARS-COVID 19 [17]. Hence, the present study aimed to measure D-Dimer levels in T2DM and COVID 19 with and without symptoms and find their correlation. In the present study, found that significantly increased levels of D-Dimer values observed in T2DM and COVID 19 with symptomatic patients when compared to asymptomatic patients (Table 1 and Figure 2). Along with that we also observed that these levels were negatively correlated with haemoglobin concentration shown in Table 2. Similarly, another study conducted with 560 subjects with COVID 19, they observed 260 subjects only the D-Dimer levels were increased and also, they reported significantly elevated levels of D-Dimer can be used for to prognostic marker for COVID 19 [18]. Previous studies also found that high levels of D-Dimer increased risk of the COVID 19 disease and mortality. Additionally, some recent studies were reported there was a significant association between the elevation of D-Dimer and severity and outcome of the COVID 19 [19-20, 21]. The present study also found the similar results and supports that previous study; there was a significant positive association between the D-Dimer and COVID 19 disease. While the global pandemic of COVID commenced in 2019, DM has been reported as being among of the significant comorbidities associated with cases of COVID-19 severe variants. Multiple research studies revealed that the prevalence of DM was around to 10%, and that individuals with severe cases had an incidence that was roughly twice that of patients without severe instances [22-23, 24]. Hyper-glycemia in patients with T2DM is thought to impair the immune system in a number of ways, including by changing macrophage function and reducing neutrophil formation, which may make it difficult for diabetic patients to prevent the spread of pathogens from outside their bodies [25]. Consequently, it is recognized that people with diabetes are more prone to infections. This lead to production of free radicals or reactive oxygen species that will damage endothelium, oxidative stress and inflammation lead to thrombosis Since there is a disparity between anticoagulation, pro-coagulation, and fibrinolysis. Recent research studies are reported that type 2 diabetic subjects are more prone to get COVID 19 disease [26]. Similarly, our study also found significantly elevated blood sugars and it was negatively correlated with CT values of COVID 19 infection. Additionally, we also determined glycated haemoglobin, and observed significantly elevated and positively correlated with COVID 19 infection. The novel SARS-COVID 19 was identified in pancreatic cells and due to infection and damage of endocrine part of beta cells of pancreas lead to insulin resistance. Insulin resistance is major risk factor for hyper-glycemia, this lead to formation of thrombus. In our study, the PPBS, HbA1c was positively correlated with D-Dimer and also, we observed there was a negative correlation between FBS and D-Dimer [27- 28]. Many investigations have found that among subjects infected with COVID-19, there is advancement in impaired liver functions. Along with that another recent researchers are reported that many of COVID 19 disease patients are observed abnormal liver function tests during the infection stage [29- 30]. The pathophysiology of liver damage in COVID 19 patients, one of the main reasons is angiotensin converting enzyme II, act as a host for novel coronavirus into the liver cells, along with this inflammatory and pro-inflammatory cytokines damage the pulmonary and extra-pulmonary cells along with the liver [31- 32]. Additionally, different types of continuous induced drugs in patients with COVID 19, also damage liver cells. Many of researchers observed increased levels of total bilirubin, direct bilirubin, AST, ALT, ALP and decreased levels of total protein and albumin [33-34]. Similarly, the present study also found significantly elevated levels of total bilirubin, direct bilirubin, AST, ALT, ALP and decreased levels in 2DM and COVID 19 patients with symptomatic when compared to asymptomatic patients. The haemoglobin levels also decreased in both the groups of study subjects and this level was negatively correlated with D-Dimer. Decreased levels of haemoglobin, albumin, and increased levels of blood sugars, HbA1c, total bilirubin, direct bilirubin, AST, ALT, ALP and D-Dimer in patients with T2DM with COVID 19, might be there is an adverse effect of this novel coronavirus. This virus not only to highly infectious and transmissible, and it will also cause to damage multi organ system.

## Conclusion:

Data shows that D-Dimer is a potential prognostic marker for COVID 19 patients with T2DM.

## Figures and Tables

**Table 1:** Comparison of Biochemical, D-Dimer and CT values between the study subjects

**Parameter**	**Group**	**Mean**	**Standard Deviation**	**P-Value**
Age (Years)	Asymptomatic	51.93	16.9	
	Symptomatic	49.42	15.63	0.277
FBS (mg/dL)	Asymptomatic	199.7	39.88	
	Symptomatic	155.45	32.21	0.001**
PPBS (mg/dL)	Asymptomatic	280.67	55.06	
	Symptomatic	286.87	69.63	0.486
HbA1c (%)	Asymptomatic	7.84	1.15	
	Symptomatic	9.33	3.17	0.001**
CT	Asymptomatic	14.22	5.59	
	Symptomatic	22.08	5.4	0.001**
D-Dimer (ng/mL)	Asymptomatic	933.64	472.66	
	Symptomatic	1727.98	325.38	0.001**
HB (g/dL)	Asymptomatic	7.44	1.38	
	Symptomatic	8.93	2.31	0.001**
T.Bil (mg/dL)	Asymptomatic	1.09	0.4	
	Symptomatic	4.37	0.73	0.001**
D.Bil (mg/dL)	Asymptomatic	0.25	0.15	
	Symptomatic	1.66	0.59	0.001**
I,Bil (mg/dL)	Asymptomatic	0.84	0.4	
	Symptomatic	2.72	0.79	0.001**
AST (IU/L)	Asymptomatic	34.18	9.52	
	Symptomatic	66.96	17.05	0.001**
ALT (IU/L)	Asymptomatic	27.11	9.1	
	Symptomatic	107.02	48.34	0.001**
ALP (IU/L)	Asymptomatic	95.01	33.68	
	Symptomatic	180.72	39.24	0.001**
Total Protein (g/dL)	Asymptomatic	7.01	0.83	
	Symptomatic	6.66	0.93	0.005*
Albumin (g/dL)	Asymptomatic	4.3	0.78	
	Symptomatic	3.49	0.77	0.001**

**Table 2:** Pearson correlation analysis of CT with biochemical, Hb and D-Dimer

**Parameter**	**CT**	
	**r**	**P Value**
FBS (mg/dL)	-0.344	0.001**
PPBS (mg/dL)	0.555	0.43
HbA1c (%)	0.234	0.001**
D-Dimer (ng/mL)	0.4	0.001**
Hb (g/dL)	0.171	0.01*
T.Bil (mg/dL)	0.569	0.001**
D.Bil (mg/dL)	0.518	0.001**
I,Bil (mg/dL)	0.504	0.001**
AST (IU/L)	0.426	0.001**
ALT (IU/L)	0.464	0.001**
ALP (IU/L)	0.468	0.001**
Total Protein (g/dL)	0.044	0.53
Albumin (g/dL)	-0.044	0.01*

**Table 3:** Pearson correlation analysis of D-Dimer with biochemical, Hb and CT

**Parameter**	**D-Dimer**	
	**r**	**P Value**
FBS (mg/dL)	-0.44	0.001**
PPBS (mg/dL)	0.977	0.02*
HbA1c (%)	0.281	0.001**
CT	0.4	0.001**
Hb (g/dL)	-0.208	0.03*
T.Bil (mg/dL)	0.686	0.001**
D.Bil (mg/dL)	0.649	0.001**
I,Bil (mg/dL)	0.588	0.001**
AST (IU/L)	0.537	0.001**
ALT (IU/L)	0.558	0.001**
ALP (IU/L)	0.518	0.001**
Total Protein (g/dL)	-0.143	0.04*
Albumin (g/dL)	-0.356	0.001*

**Table 4:** Pearson correlation analysis of Hb values with biochemical, D-Dimer and CT

**Parameter**	**Hb**	
	**r**	**P Value**
FBS (mg/dL)	-0.202	0.04*
PPBS (mg/dL)	-0.415	0.41
HbA1c (%)	0.11	0.12
CT	0.171	0.01**
D-Dimer (ng/mL)	-0.208	0.03*
T.Bil (mg/dL)	0.306	0.001**
D.Bil (mg/dL)	0.229	0.01*
I,Bil (mg/dL)	0.306	0.001**
AST (IU/L)	0.392	0.001**
ALT (IU/L)	0.247	0.001**
ALP (IU/L)	0.295	0.001**
Total Protein (g/dL)	-0.36	0.06
Albumin (g/dL)	-0.179	0.01*
